# Clinical and inheritance profiles of Kallmann syndrome in Jordan

**DOI:** 10.1186/1742-4755-1-5

**Published:** 2004-10-24

**Authors:** Mousa A AbuJbara, Hanan A Hamamy, Nadim S Jarrah, Nadima S Shegem, Kamel M Ajlouni

**Affiliations:** 1 The National Center for Diabetes, Endocrinology and Genetics, Amman, Jordan

**Keywords:** Kallmann syndrome, Hypogonadotropic hypogonadism, Microphallus, Jordan

## Abstract

**Background:**

Proper management of patients with Kallmann syndrome (KS) allows them to attain a normal reproductive health. The purpose of this study is to demonstrate the presentation modalities, phenotypes and the modes of inheritance among 32 patients with Kallmann syndrome in Jordan. Recognition of the syndrome allows for prompt proper management and provision of genetic counselling.

**Subjects:**

Over a period of five years (1999–2004), the clinical and inheritance profiles of 26 male and 6 female patients with Kallmann syndrome from 12 families were evaluated at the National Center for Diabetes, Endocrinology and Genetics in Jordan.

**Results:**

The patients belonged to twelve Jordanian and Palestinian families and their age at presentation ranged from 4 – 46 years. Nine boys aged 4–14 years presented with cryptorchidism and microphallus, all other males presented with delayed puberty, hypogonadism and/or infertility. The main presentation among six female patients was primary amenorrhea. Intrafamilial variability in clinical phenotype was specifically evident for renal abnormalities and sensorineural hearing impairment. Familial KS was diagnosed in 27 patients belonging to five families with the X-linked mode of inheritance and two families with the autosomal recessive mode of inheritance.

**Conclusions:**

(1) the majority of cases in this study represented the X-linked form of KS, which might point to a high prevalence of Kal 1 gene in the population. (2) Genetic counselling helps these families to reach a diagnosis at an early age and to decide about their reproductive options. (3) Children presenting with cryptorchidism and microphallus in our population should be investigated for KS.

## Background

One of the most common causes of hypogonadotropic hypogonadism is Kallmann syndrome (KS). KS is a genetically heterogeneous condition that affects approximately one in 8000 males and one in 40,000–70,000 females [1-3]. This recent estimate is much higher than the previously estimated prevalence of Kallmann syndrome among males of 1:80,000 [4].

To our knowledge, no data is available on the incidence of this syndrome in Jordan or in the Arab world.

In addition to the sporadic form which is the most common [2], KS has three modes of inheritance, X-linked, autosomal recessive and autosomal dominant [5]. The gene responsible for the X-linked form of the disease is KAL1 gene [6-8], and encodes the protein anosmin that is directly responsible for the migration of GnRH neurons and the olfactory nerves from the olfactory system to the hypothalamus [5,9-11]. Males usually present in the second decade with delayed puberty and females present with primary amenorrhea. Prepubertal boys may present with microphallus and cryptorchidism [12,13]. Proper management of patients with Kallmann syndrome usually allows them to attain normal reproductive health. The purpose of this paper is to demonstrate the presentation modalities, phenotypes and the modes of inheritance among 32 patients with Kallmann syndrome in Jordan, which may contribute to the recognition of the syndrome and the provision of adequate management and genetic counselling.

## Subjects & Methods

Over a period of five years, thirty-two male and female patients from twelve Jordanian and Palestinian families were referred to the National Center for Diabetes, Endocrinology and Genetics (NCDEG) in Amman, Jordan for evaluation of hypogonadism among adults, or microphallus among children.

**Figure 1:**
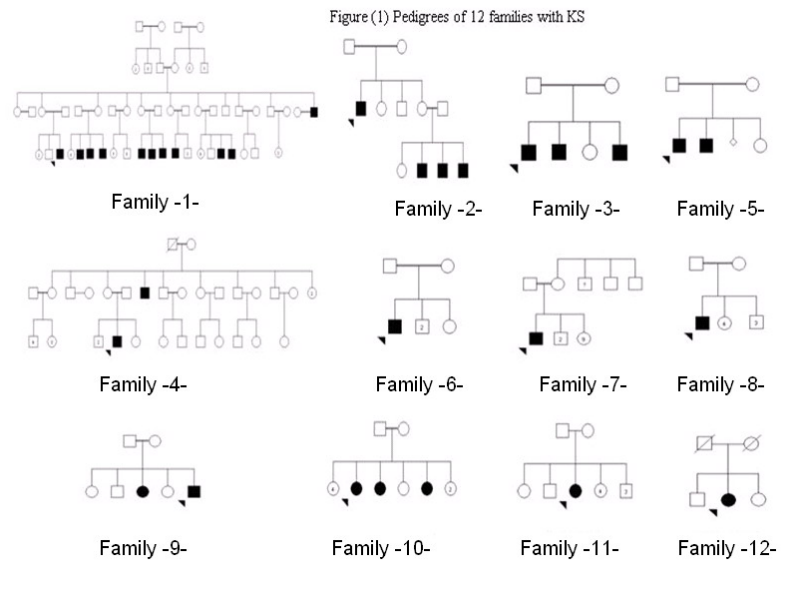
Pedigrees of 12 families with KS

Prospective evaluation was performed including pedigree construction (fig 1), and complete clinical examination with special emphasis on assessment of anosmia, the presence of mirror image movements (synkinesia) and examination of external genitalia.

Olfactory testing was performed through a smell identification test designed by our department using odours that can be easily identified among all social classes in the local population. These odours included cinnamon, coffee, camomile, thyme, soap, oriental perfume and tobacco. The specific odoriferous substance was approximated to the nostrils while the patient had his eyes closed. The patient is then asked to name the olfactory stimulus. According to the number of substances identified by smelling, patients were divided into anosmic and hyposmic. Those who failed to identify all odours were categorized as anosmic, while those who identified three or less odours were categorized as hyposmic. Those who identified four to all seven odours were considered normosmic. First-degree relatives of the probands were also questioned and tested for the smell sensation in a similar fashion. The same investigator carried out olfactory testing for all patients and family members.

In addition to the assessment of smell sensation among relatives of probands, their answers to specific questions were recorded. These questions addressed the issues of surgery to correct cryptorchidism, administration of testosterone and whether there were any complaints of delayed puberty or infertility. The specific questions and the olfactory testing led to the identification of further cases of KS in the family.

Patients were asked to perform a screwdriver motion in one hand while the examiner watches for any similar non-voluntary movement in the other hand to assess mirror image movements. Subjects were also asked to perform alternate supination and pronation of one forearm while the examiner watches for a similar movement in the other arm [14]. Phallus length was measured and compared to age-matched controls [15], and the testicle size was estimated using an orchidometer. Neurosensory hearing impairment was assessed by audiometry, renal abnormalities by renal ultrasound and congenital heart defects by echocardiography. All patients were tested for color vision and abnormal eye movements. Complete hormonal evaluation including basal gonadotrophins, sex hormones and gonadotrophin releasing hormone (GnRH) stimulation test were done for 20 patients 16 years and above. Three adult patients were not tested due to loss of contact. GnRH test involved the administration of 100 mcg of GnRH, followed by the assessment of gonadotrophin levels at 0, 15, 30, 45, 60 and 120 minutes. For individuals with no or low response to the test, priming was performed, where GnRH was given for five days, with repetition of assessment of gonadotrophin levels. Seminal fluid analysis was done for male patients aged 16 years and above. Radiological studies included brain, pituitary and olfactory tract magnetic resonance imaging (MRI).

The criteria for diagnosis of KS among adults included the presence of anosmia or hyposmia with clinical signs and symptoms of hypogonadism and a testosterone level <100 ng/dl among males 16 years and older, and estradiol level <20 pg/dl among adult females, together with basal low gonadotrophin level. In family II, loss of contact of three brothers hindered testing their hormonal levels. They were, however, included among our patients because clinical examination revealed hypogonadism, anosmia and micropenis. Among prepubertal males, the criteria for diagnosis of KS included presence of microphallus with anosmia/hyposmia, and/or absent olfactory bulbs on MRI.

Pedigree analysis was used to establish the modes of inheritance of KS in the familial cases. Inheritance in a family was classified as X-linked if only males were affected in more than one sibship connected by females, or if two or more males were affected in the sibship with a negative family history and with associated synkinesia. Inheritance was classified as autosomal recessive if all affected individuals were members of the same generation and included at least one female.

## Results

Over a period of five years (1999–2004), thirty-two patients were prospectively diagnosed with Kallmann syndrome at the National Center for Diabetes, Endocrinology and Genetics (NCDEG) in Amman, Jordan. The patients belonged to twelve Jordanian and Palestinian families and their ages at presentation ranged between 4 – 46 years. They included 26 males and 6 females with a male/female ratio of 4.34/1. Nine male patients were aged 14 years and younger.

The clinical features among male patients are presented in table (1), and among female patients in table (2).

**Table 1:** Clinical features of 26 males with Kallmann syndrome

FAMILY	No	Age	Anosmia/hyposmia	Synkinesia	Hearing Impairment	Renal abnormalities	Azoospermia	cryptorchidism	Micropenis
**1**	1	46	+/H	-	+	-	+	+	-
	2	14	+	+	+	+	+	+	-
	3	27	+	+	-	+	+	+	-
	4	20	+	+	-	+	+	+	-
	5	20	+	+	-	+	+	+	-
	6	19	+	+	-	+	+	+	-
	7	16	+	+	-	+	+	+	-
	8	14	+	+	-	+	NA	+	-
	9	9	+	+	-	+	NA	+	+
	10	6	+	+	-	-	NA	-	+
	11	4	+	+	-	-	NA	+	+
**II**	1	37	+	-	-	-	+	+	+
	2	24	+	-	ND*	ND*	ND*	+	+
	3	22	+	-	ND*	ND*	ND*	-	+
	4	20	+	-	ND*	ND*	ND*	+	+
**III**	1	14	+	+	-	-	NA	-	+
	2	10	+	+	-	+	NA	-	+
	3	8	+	+	-	+	NA	-	+
**IV**	1	6	+	+	-	-	NA	+	+
	2	5	+	+	-	-	NA	+	+
**V**	1	20	+/H	-	+	-	+	-	+
	2	19	+/H	+	+	+	+	-	+
**VI**	1	19	+	-	-	+	ND	+	-
**VII**	1	37	+	-	-	-	+	+	+
**VIII**	1	22	+	-	-	+	+	+	+
**IX**	1	20	+	-	-	-	oligospermia	+	+

NA: not applicable.

ND: not done.

*: lost contact

H: hyposmia

**Table 2:** Clinical Features of 6 females with Kallmann syndrome

FAMILY	No	Age	Anosmia/hyposmia	Synkinesia	Hearing loss	Renal abnormalities	Primary Amenorrhea
**X**	1	23	+	-	-	-	+
	2	21	+	-	-	-	+
	3	18	+	-	-	-	+
**IX**	1	18	+/H	-	-	-	+
**XI**	1	30	+	-	-	-	+
**XII**	1	18	+/H	-	-	-	+

H: hyposmia

**Table 3:** Hormonal profile and GnRH stimulation test in adult male patients with KS.

FAMILY		Age	Inheritance	Base line LH (mIU/ml)	Peak LH (mIU/ml) after GnRH testing	Peak LH (mIU/ml) after priming	Baseline FSH (mIU/ml)	Peak FSH (mIU/ml) after GnRH testing	Peak FSH (mIU/ml) after priming	Testosterone ng/dl
**1**	1	46	XR	0.5	2.26		1.09	2.22		60
	2	14	XR	Undetected	0.35	2.50	0.64	2.39	4.25	Undetected
	3	27	XR	Undetected	0.90	2.85	0.59	1.58	5.24	Undetected
	4	20	XR	0.6	2.90		1.66	3.43		80
	5	20	XR	0.8	3.10		2.1	5.6		50
	6	19	XR	Undetected	0.40	2.88	0.8	2.3	6.4	Undetected
	7	16	XR	0.6	3..50		1.35	3.21		50
**II**	1	37	XR	Undetected	0.50	3.25	0.43	2.14	6.34	Undetected
	2	24	XR	ND	ND	ND	ND	ND	ND	ND
	3	22	XR	ND	ND	ND	ND	ND	ND	ND
	4	20	XR	ND	ND	ND	ND	ND	ND	ND
**V**	1	20	XR	Undetected	0.45	2.1	0.47	1.65	3.2	Undetected
	2	19	XR	0.42	4.36		0.90	5.22		40
**VI**	1	19	sporadic	0.5	6.2		0.9	10.1		70
**VII**	1	37	sporadic	Undetected	1.9		0.46	3.2		Undetected
**VIII**	1	22	sporadic	0.53	3.25		0.85	4.50		Undetected
**IX**	1	20	AR	0.85	4.25		1.21	6.30		50

ND: not done (lost contact)

XR :X-linked recessive

AR :autosomal recessive

**Table 4:** Hormonal profile and GnRH stimulation test in adult female patients with KS.

FAMILY		Age	Inheritance	Base line LH (mIU/ml) N=	Peak LH (mIU/ml) after GnRH testing	Peak LH (mIU/ml) after priming	Base line FSH (mIU/ml) N=	Peak FSH (mIU/ml) After GnRH testing	Peak FSH (mIU/ml) after priming	Estradiol Pg/ml
X	1	23	AR	0.6	7.25		2.6	19.33		5
	2	21	AR	0.5	3.5		1.95	6.60		7
	3	18	AR	0.6	5.2		1.2	9.5		10
IX	1	18	sporadic	0.8	5.10		2.1	5.6		15
XI	1	30	sporadic	0.7	4.2		1.5	6.2		10
XII	1	18	sporadic	Undetected	2.10		2.1	5.8		Undetected

AR :autosomal recessive

Twenty seven patients were anosmic and five patients were hyposmic (table 1). In family V, both affected brothers were hyposmic.

Cryptorchidism was found or previously operated on in 19/26 (73%) and microphallus in 17/26 (65%) male patients respectively. All patients included in this study manifested high-arched palate. Renal abnormalities including unilateral renal agenesis, malrotated kidney, and horseshoe kidney were detected in 11/19 (58%)cases with the X-linked form of KS. Two sporadic cases showed renal anomalies. A variable degree of sensorineural hearing impairment was found in 4/19 patients with X-linked KS, and in none of the other mode of inheritance or the sporadic cases. Olfactory MRI revealed olfactory tract agenesis among 19/24 cases for which the investigation was done in the series.

Among females diagnosed as KS in this series primary amenorrhea was the main presenting feature.

Pedigrees were constructed for all families (figure 1). Five cases were sporadic and 27 cases were familial belonging to seven families. Pedigree analysis assigned an X-linked mode of inheritance to 3 families with affected males linked through normal females (families I, II and IV). Family I is the largest family in our series with 11 affected males. Two further families were designated as having the X-linked form of KS because the affected males among these siblings displayed synkinesia with a negative family history (families III and V). Synkinesia has been reported to be associated only with the X-linked form of KS [16]. However, a recent report identifying the specific gene mutated in autosomal dominant KS pointed out that synkinesia may occur in the autosomal forms of KS [17]. In families III and V in this report, pedigree analysis strongly pointed to the X-linked form of KS although autosomal recessive inheritance cannot be definitely excluded. Two families were designated as having the autosomal recessive mode of inheritance. One family included affected brother and sister with normal consanguineous parents (family IX), while the other family had 3 affected sisters with normal consanguineous parents (family X). Consanguinity rate among parents of all patients was 83%, with 50% of all marriages being between first cousins.

## Discussion

This study points to the higher proportion of the X-linked form of Kallmann syndrome among all KS cases seen at an endocrine/genetic clinic in Jordan over a period of 5 years. Among 7 families with inherited KS, the X-linked form was the mode of inheritance in 5 families (71% of familial KS). None of the pedigrees was consistent with autosomal dominant inheritance in this series. In the two families with autosomal recessive inheritance, the probability of a non-penetrant autosomal dominant gene in either parent was considered remote because of absence of any relevant family history. Autosomal dominant inheritance was also considered a very remote possibility in the X-linked families because of absence of affected individuals in earlier generations and the pattern of inheritance as revealed by pedigree construction (fig 1) was compatible with X-linked inheritance. The X-linked form of KS has at times been reported to account for only one third of inherited cases [1], and at other times to be the most frequent form [2]. The high proportion of the X-linked form among our cases may represent a high prevalence of Kal1 gene among Jordanians and Palestinians. Consanguineous marriages in Jordan are favored culturally. Among two thousand marriages in the general population, 32% have been reported to be between first cousins [18]. The figure of 50% first cousin marriages among parents of our patients would thus reflect the high consanguinity rate among the population in general.

Among the XR form of KS, 75% of patients were reported to show synkinesia [9]. Synkinesia in X-linked KS has been attributed to an abnormal projection of the corticospinal tract [19]. In our experience, synkinesia was present in 16/22 (73%) patients with the XR form of KS and was characteristically more pronounced in the younger age group. Intrafamilial clinical heterogeneity has been reported among family members carrying the same mutation in Kal 1 gene [9]. In this series, intrafamilial variability in renal anomalies was exemplified in family I, the largest family in our series, where eight out of eleven patients had renal abnormalities. Intrafamilial variability was also seen in family III, in which one patient had a malrotated kidney, his brother had a horseshoe kidney while the third brother had no renal anomalies.

Sensorineural hearing loss has also been reported to be associated mainly with the X-linked form of KS [20]. The KAL1 gene is expressed in the inner ear from early developmental stages suggesting that the defect underlying the hearing loss in X-linked Kallmann syndrome occurs during the organogenesis period [9]. In our study, sensorineural hearing impairment was only diagnosed among patients with the X linked form of Kallmann syndrome. Four of nineteen tested males (21%) showed sensorineural hearing impairment with evident intrafamilial variability.

In this series cryptorchidism or a history of cryptorchidism was present in 73% of patients (19/26), and was not related to a specific mode of inheritance or etiology. Microphallus was present among 17/26 (65%) patients in this study, with several other patients reporting a history of treatment with testosterone; the exact number of treated patients or the treatment profile could not be precisely determined. Seminal fluid analysis was done for all patients of 16 years and above. The test showed azoospermia for all tested males except patient 1 in family IX, where oligospermia was reported with a sperm count of 10,000,000 per ml. None of the patients with the X-linked form manifested ichthyosis, mental retardation, short stature or ocular albinism, pointing to the underlying etiology being a mutation in the Kal 1 gene rather than a contiguous-gene deletion syndrome [21].

Olfactory MRI revealed olfactory tract agenesis in 80% of cases for which the investigation was done in the series (19/24). Quinton et al, 1996, indicate that KS may be present with no pathology detected in the olfactory tract on MRI, and that phenotypic characterization of KS was effectively achieved by accurate estimation of olfactory sensation [22]. Kallmann syndrome has a favorable prognosis under proper management. Its investigation should thus be considered in any child presenting with cryptorchidism and microphallus. Since the gonad state is still dormant in childhood, gonadotrophin levels are not helpful. Olfactory MRI may be a more useful tool for the diagnosis [23]. Nevertheless, a normal MRI does not rule out Kallmann syndrome as normal olfactory bulbs can be present in up to 25% of cases [22]. The presence of anosmia/hyposmia and history of delayed puberty or infertility in the family are helpful in establishing the diagnosis. Where diagnosis remains difficult, it is indicated to follow up these children till they reach puberty.

The majority of Kallmann syndrome cases in our study showed the X-linked mode of inheritance, which might indicate a high prevalence of Kal1 gene in the population. However, molecular studies for the Kal1 gene were not performed in this study. Patients in our series manifested a wide range of phenotypic heterogeneity with intrafamilial variability of clinical manifestations. We recommend an evaluation for Kallmann syndrome in our population in any child presenting with microphallus and cryptorchidism. Further studies are needed to establish the prevalence rate of Kallmann syndrome in Jordan and to define the causative mutations.

## Competing interests

The authors declare that they have no competing interests.

## Authors' contributions

MAJ and KA were the main researchers. HH and MAJ drafted the manuscript. All authors were part of the team that evaluated the patients
